# Relationship Between Vitamin D Receptor Gene BsmI Polymorphism and Fibromyalgia Syndrome

**DOI:** 10.7759/cureus.27113

**Published:** 2022-07-21

**Authors:** Sidrah Parvez, Ghizal Fatima, Farzana Mehdi, Najah R Hadi, Jan Fedacko

**Affiliations:** 1 Department of Biotechnology, Era's Lucknow Medical College and Hospital, Lucknow, IND; 2 Department of Personalized and Molecular Medicine, Era's Lucknow Medical College and Hospital, Lucknow, IND; 3 Department of Pharmacology and Therapeutics, University of Kufa, Najaf, IRQ; 4 Department of Cardiology, Pavol Jozef Šafárik University, Kosice, SVK

**Keywords:** rflp, pcr, vitamin-d receptor, bsmi polymorphism, fibromyalgia

## Abstract

Purpose

Vitamin D receptor (VDR) has been proposed as a possible marker for fibromyalgia syndrome (FMS). The purpose of this study is to characterize the expression pattern of BsmI polymorphism (rs1544410) in the VDR gene in women with FMS and the genotype-phenotype association.

Methods

A total of 105 FMS patients and 105 controls were included in this study. VDR gene BsmI polymorphism was assessed by polymerase chain reaction (PCR) and restriction fragment length polymerase (RFLP) method.

Results

There was no significant difference in the frequency distribution of both genotypes and alleles for VDR gene BsmI polymorphism between FMS patients and controls (p>0.05). The frequencies of BB, Bb, and bb in the VDR gene BsmI polymorphism were 19%, 43%, and 37% in patients, while in controls were 22.9%, 55.2%, and 21.9%. However, we did not find any significant association between the clinical symptoms of this disease and VDR BsmI genotypes among FMS patients (p>0.05).

Conclusions

The relationship between the VDR gene BsmI polymorphism and FMS could not be determined in this study. However, further studies with a larger sample size may be required to show a relation between the VDR gene BsmI polymorphism and FMS.

## Introduction

Fibromyalgia syndrome (FMS) is a widespread chronic musculoskeletal pain disorder characterized by symptoms like morning stiffness, fatigue, multiple tender points, sleep disorder, headache, low pain threshold, anxiety, and depression [1]. The incidence of FMS occurs from 0.2 to 6.6 percent in the general population and 2.4 to 6.8 percent in women [2]. Females are nine times more likely to develop FMS compared to men, according to research [3]. The origin and aetiology of FMS are unclear, and the causes of chronic widespread pain in FMS patients are also unknown. Pain in FMS may be influenced by factors such as autonomic and neuroendocrine disorders, as well as acute sensitization processes [4]. FMS is thought to be caused by a variety of factors, including neurological and autonomic nerve system issues, psychological disorders, as well as genetic and environmental factors [5]. FMS is a multi-factorial disorder with a hereditary vulnerability. This hereditary vulnerability, when combined with environmental factors such as psychological and emotional stressors, raises the risk of symptoms causing and exacerbating FMS [6]. FMS has shown both familial aggregation and genetic polymorphism, indicating that it is a genetic predisposition. Both FMS patients and healthy people had specific genes that helped to analyze the amplitude of specific genetic polymorphisms. Recent studies have shown that polymorphisms in genes associated with the serotoninergic, dopaminergic, and catecholaminergic systems contribute to the aetiology of FMS [7].

The action of vitamin D receptor (VDR) and the enzyme-mediated activity of 1-hydroxylase in many areas of the central nervous system, especially in the hypothalamus, may contribute to the central sensitization which is responsible for the development of chronic widespread pain in FMS. It has been proposed that VDR and the 1-hydroxylase may contribute to the triggering of chronic headaches and migraines [8]. Vitamin D is important for dopamine and serotonin release, which may be considered as neurotransmitters in the aetiology of FMS [9]. The VDR gene is found on 12 cen-q12 chromosomes. It contains 12 exons and contains genomic DNA of nearly 75kb [10]. The VDR gene has reported many restriction fragment length polymorphisms (RFLPs), including sites cleaved by TaqI, BsmI, FokI, and ApaI. The allele variants of the VDR gene are FokI (F/f allele), TaqI (T/t allele), BsmI (B/b allele), and ApaI (A/a allele). The function of VDR gene polymorphisms has been reported in many chronic pain studies, such as osteoarthritis [11], lumbar pain [12], and migraine [13]. Reports are lacking regarding the influence of VDR BsmI genetic variants in women with FMS patients. To our knowledge, this is the first study investigating the association of the VDR BsmI gene polymorphism in women with FMS in the North Indian population. The purpose of this present study was to characterize the expression pattern of the VDR gene BsmI polymorphism in women with FMS and its association with its clinical symptoms.

## Materials and methods

Study population

The Research Ethics Committee of the Era's Lucknow Medical College and Hospital (ELMCH), Era University, Lucknow, India approved this study. All the procedures including human subjects were performed in accordance with the ethical standards of ELMCH, Era University, Lucknow, India. A total number of 105 FMS patients and 105 controls were involved in this case-control study.

Patients and controls selection criteria

All the FMS patients and controls were female and came from the same ethnic group and geographical region. Patients were participants of the ELMCH, Lucknow, who attended the outpatient department (OPD) from the department of rheumatology. The study includes only those patients with FMS who will fulfill the American College of Rheumatology criteria 2016 [14]. Patients with diabetes, rheumatoid arthritis, systemic lupus erythematosus, and multiple myeloma or any other endocrinology disease were excluded from the study. The mean time for the patients suffering with fibromyalgia was 19.5±2.5 weeks. The mean age of FMS patients was 36.1±11.1 years, and controls were 34.6±10.3 years. The controls were participants of the ELMCH, Lucknow, who were visiting for a routine check-up. The controls were defined as those without FMS and no past history of rheumatic disease, and not receiving any medicine at the time of enrolment in the study.

Data and sample collection

During the time of enrolment, the patients and controls were provided with questionnaires to be filled out, and the questionnaire inquired about the duration of symptoms, age, body mass index (BMI), Fibromyalgia Impact Questionnaire Revised (FIQR), pain intensity is measured by visual analog scales (VAS), weight loss, jaw pain, frequent awakening, family history, fatigue, headache, stiffness, feeling of fever, lack of energy, abdominal pain, restless leg, and irritable bowel syndrome. After signing the informed consent form, blood samples were collected from the patients and controls in tubes containing ethylene diamine tetra acetic acid (EDTA) and stored at 20°C for genetic analysis.

VDR BsmI genotyping

The peripheral blood was taken in a blood collection tube from the FMS patients and controls after taking written consent. The salting out method was used for the DNA isolation from the blood samples. Extracted DNA was used for the amplification of the VDR BsmI gene polymorphism by polymerase chain reaction-restriction fragment length polymerase (PCR-RFLP) method. The primer details for the VDR BsmI gene polymorphism were given in Table 1.

**Table 1:** Primer sequences used for VDR BsmI gene polymorphism

Primer		Sequence
VDR BsmI	Forward	5′- CAACCAAGACTACAAGTACCGCGTCAGTGA -3′
	Reverse	5′- AACCAGCGGAAGAGGTCAAGGG -3′

PCR was performed in a 25µl reaction mixture of 150-200ng genomic DNA, 25pmol of each primer, and 2X master mix (Takara, Kusatsu, Japan) per tube using a gradient MasterCycler (Bio-Rad, Hercules, California). The PCR protocol is as follows: initial denaturation at 94°C for five minutes, followed by 30 cycles of denaturation at 94°C for 40 seconds, annealing at 62°C for 40 seconds, extension at 72°C for 40 seconds, final extension at 72°C for five min. After amplification, PCR products (825bp) were digested with BsmI (New England Biolabs Inc, Ipswich, Massachusetts) restriction endonuclease at 65°C for one hour. Digested products were run on agarose gel (2%) containing ethidium bromide and visualized by a gel documentation system (EZ, Bio-Rad, Hercules, California). The absence of a BsmI restriction site was indicated as a B allele, while the presence of the BsmI site was indicated as a b allele that generates two or three fragments. The single uncut fragment of 825bp bands was indicated as BB homozygous. The bb homozygous generates two fragments of 650bp and 175bp, while the Bb heterozygous generates three fragments 825bp, 650bp, and 175bp. 

Statistical analysis

FMS patients and controls were compared using statistical analysis performed with sufficient post hoc analysis using SPSS statics 28.0 software (IBM Inc., Armonk, New York). The chi-square (χ2) test or Fisher's exact test were used to compare the genotyping data between FMS patients and controls. Odds ratio (OR), risk ratio (RR), and 95% confidence interval (CI) were used to assess the risk factors. χ2 test was used to evaluate the Hardy-Weinberg Equilibrium (HWE) for the genotype distribution of the patients and controls. The value was considered to be significant when p<0.05.

## Results

Table 2 shows the distribution frequency of genotypes and alleles of the VDR BsmI gene polymorphism in FMS patients and controls based on univariate analysis. There was no significant difference in the frequency distribution of both genotypes and alleles for the VDR gene BsmI polymorphism between FMS patients and controls. The frequencies of BB, Bb, and bb in the VDR gene BsmI polymorphism were 19%, 43%, and 37% in patients, respectively, while in controls were 22.9%, 55.2%, and 21.9%. The frequency distribution of B and b alleles was 41% and 59%, respectively and in controls were 50.5% and 49.5%.

**Table 2:** Distribution of genotype and allele frequency of VDR BsmI gene polymorphism in FMS patients and controls VDR - vitamin D receptor; FMS - fibromyalgia syndrome; p<0.05 is considered significant

Genotypes	Patients (n=105)	Controls (n=105)	p-value	χ^2^-value
BB	20 (19)	24 (22.9)	0.05	5.87
Bb	46 (43)	58 (55.2)		
bb	39 (37)	23 (21.9)		
Alleles				
B	86 (41)	106 (50.5)	0.06	3.46
b	124 (59)	104 (49.5)		

Table 3 shows the association of VDR BsmI gene polymorphism with the clinical symptoms of FMS patients. Age, BMI, FIQR, VAS, weight loss, jaw pain, frequent awakening, family history, fatigue, headache, stiffness, feeling of fever, lack of energy, abdominal pain, restless leg, and irritable bowel syndrome were analyzed. We did not find any significant association between VDR BsmI genotypes and clinical symptoms in FMS patients (p>0.05).

**Table 3:** Association of VDR BsmI gene polymorphism with the clinical symptoms of FMS patients VDR - vitamin D receptor; FMS - fibromyalgia syndrome; SD - standard deviation; BMI - body mass index; FIQR - Fibromyalgia Impact Questionnaire Revised; VAS - visual analog scales; p<0.05 is considered significant

Characteristics	BB (n=20)	Bb (n=46)	bb (n=39)	p-value
Age, Mean±SD	37.2±11.5	37.1±11	33.8±11.0	0.34
BMI, Mean±SD	24.1±3.9	25.4±4.0	23.9±4.3	0.21
FIQR, Mean±SD	74.2±0.8	76.7±2.0	75.4±5.9	0.06
Pain (VAS), Mean±SD	4.1±1.2	3.8±0.4	4.2±0.8	0.11
Weight loss, n (%)	5 (21.7)	7 (30.4)	11 (47.8)	0.32
Jaw pain, n (%)	8 (25.0)	9 (28.1)	15 (46.9)	0.09
Frequent awakening, n (%)	18 (18.9)	40 (42.1)	37 (38.9)	0.46
Family history, n (%)	9 (15)	23 (38.3)	28 (46.7)	0.06
Fatigue, n (%)	18 (18.9)	44 (46.3)	33 (34.7)	0.22
Headache, n (%)	9 (19.1)	15 (31.9)	23 (48.9)	0.05
Stiffness, n (%)	14 (17.5)	39 (48.8)	27 (33.8)	0.18
Feeling of fever, n (%)	15 (16.7)	43 (47.8)	32 (35.6)	0.10
Lack of energy, n (%)	14 (17.9)	38 (48.7)	26 (33.3)	0.21
Abdominal pain, n (%)	17 (19.1)	42 (47.2)	30 (33.7)	0.18
Restless leg, n (%)	11 (19.3)	21 (36.8)	25 (43.9)	0.23
Irritable bowel syndrome, n (%)	12 (17.4)	26 (37.7)	31 (44.9)	0.07

Table 4 represents the frequency distribution of VDR BsmI genotypes in FMS patients and controls using logistic regression analysis. Except for the recessive model, none of the other models show any significant differences between FMS patients and controls. In the recessive model, bb genotypes was found higher in FMS patients than controls (37% vs. 20%; p=0.009; OR=0.42, 95%CI=0.22-0.78; RR = 0.67, 95%CI=0.52-0.87).

**Table 4:** Comparison of VDR BsmI genotype among FMS patients and controls by logistic regression analysis VDR - vitamin D receptor; FMS - fibromyalgia syndrome; OR - odds ratio; RR - risk ratio; CI - confidence interval; p<0.05 is considered significant

Genotype	Patients (n=105)	Controls (n=105)	OR (95% CI)	RR	p-value
Co-dominant model					
BB	20 (19)	24 (22.85)	-	-	-
Bb	46 (43)	58 (55.2)	1.05 (0.51-2.1)	1.02 (0.69-1.51)	0.89
bb	39 (37)	23 (21.9)	0.49 (0.22-1.07)	0.69 (0.48-1.01)	0.11
Dominant model					
BB	20 (19)	24 (22.85)	-	-	-
Bb+bb	85 (81)	81 (77.1)	0.79 (0.40-1.54)	0.88 (0.62-1.26)	0.61
Recessive model					
BB+Bb	66 (62.9)	84 (80)	-	-	-
bb	39 (37)	21 (20)	0.42 (0.22-0.78)	0.67 (0.52-0.87)	0.009

## Discussion

The assumption is that environmental and genetic multi-factors play a significant role in FMS aetiology, as shown by numerous polymorphisms. According to several studies, genetic predispositions have a significant influence on the widespread development of chronic pain in FMS. FMS can occur in individuals with a genetic vulnerability due to environmental variables and/or the pathophysiology of neuroendocrine and autonomous abnormalities [4,15-16]. Studies of family aggregation and its association with certain gene polymorphisms in FMS have received considerable attention in recent years [17]. Dopaminergic, catecholaminergic, serotonergic, and apolipoprotein E studies have revealed that genetic variants have a role in etiopathogenesis [1,18]. However, there are various studies depicting both the positive and negative correlations of vitamin D in FMS patients [19].

Vitamin D is necessary for calcium absorption. Vitamin D works by regulating gene expression when it binds to VDR. Potential genetic variations occur in specific genes after vitamin D exposure, which may modify bioavailability, transitions, and distribution in the pool for lipid accumulation, retention, and vitamin D action [20]. Vitamin D can cause pain through various mechanisms. Vitamin D's role in neuronal cells, along with the 1-hydroxylase complex and VDR, which trigger pain via cytokine-mediated pathways, is also thought to enhance central sensitization [21-22]. The purpose of this study was to determine the VDR gene polymorphism role in the FMS aetiology. Although the role of the VDR gene is implicated in chronic pain conditions such as migraine [23], osteoarthritis [24], and lower back pain [25], its significance in central hypersensitization-mediated pain pathways is unknown. Many polymorphisms have been reported in the VDR gene. FokI, TaqI, ApaI, and BsmI are the most investigated polymorphism [26].

To the best of our knowledge, no previous studies have examined the association of the VDR gene BsmI polymorphism in North Indian women with FMS. There was no statistically significant difference between the genotypes and alleles frequency distribution of the VDR gene BsmI polymorphism in the FMS patients and controls. Our results are in agreement with other studies. Santos et al. observed that no significant differences in the distribution of the genotypes and alleles frequency of the VDR gene BsmI polymorphism among FMS patients and controls in Brazilian women (p=0.06 and p=0.07) [27]. In addition, we did not find any critical interaction between the VDR gene BsmI polymorphism and clinical symptoms among FMS patients (p>0.05) in this study.

Other authors found no association between the VDR gene BsmI polymorphism in postmenopausal women with osteoporosis [28] and obese Egyptian male-medical students [29]. A case-control study in the Kashmiri population has reported a significant association of VDR gene BsmI polymorphism with temporomandibular joint disorders [30].

The limitation of this study was the evaluation of only the VDR BsmI gene polymorphism in FMS. The relation between the VDR BsmI gene polymorphism and FMS could be better understood in the future by examining BsmI polymorphism in a larger sample size. Therefore, more accurate results can be obtained between the VDR BsmI gene polymorphism and FMS.

## Conclusions

Our study is the first report of VDR BsmI genetic polymorphism in North Indian women with FMS. No significant results were observed between the VDR BsmI gene polymorphism and FMS. Thus, we could say that VDR BsmI gene polymorphism is not an important contributor for FMS in the North Indian ethnic group.
